# Visual Diagnosis: Pearling: a case study

**DOI:** 10.1186/1865-1380-4-74

**Published:** 2011-12-08

**Authors:** David P Nguyen, Bobby K Desai, Michael Falgiani

**Affiliations:** 1 Department of Emergency Medicine, University of Florida, PO Box 100186, Gainesville 32610, FL, USA

## Abstract

We present the case of a patient who attempted to perform a type of body modification known as "pearling" or "genital beading" while in prison. This patient unfortunately caused severe trauma to his penis, requiring surgical intervention. Photographs of the traumatic injuries are presented.

## Background

"Pearling," also known as "genital beading" is the practice of permanently inserting small beads made of various materials beneath the skin of the genitals [1]. As well as being an aesthetic practice, this is usually intended to enhance the pleasure of partners during sexual intercourse by increasing physical stimulation. It is most commonly done on the dorsal surface of the shaft of the penis where small, superficial incisions are made and beads are placed under the skin surface. Most implants are made of small inert metal beads (stainless steel, titanium) or plastic beads (nylon, silicone).

This form of body modification is still practiced in various world cultures. Historically, the Yakuza of Japan, an organized crime syndicate, is the most well known for "pearling." Each pearl supposedly symbolizes each year that was spent in prison. Interestingly, "pearling" has become more commonplace in the United States, especially in the US prison system.

## Case presentation

A 19-year-old male inmate presented to our Emergency Department (ED) after attempting to purposefully cut the dorsal surface of his penis with a brand-new razor blade for self-performed "pearling." He made two horizontal incisions on the shaft, one proximal and close to the base of the penis, and one distal near the glans penis. This was performed approximately 6-7 h prior to arrival at the ED. The patient alerted the prison staff to request medical evaluation after he noted worsening pain, swelling and ecchymosis to his penis, as well as a significant amount of blood when urinating. Upon arrival, the patient appeared to be in no acute distress, without obvious active bleeding. He denied dysuria.

In the Emergency Department, the patient's initial vital signs were: temperature of 37°C, pulse of 84 beats per minute, respiratory rate of 16, and blood pressure of 141/88 mmHg. His airway was patent with clear, bilateral breath sounds and unlabored breathing. On cardiac exam the patient had a regular rate and rhythm. His abdomen was soft, non-tender, and non-distended. Neurological exam revealed no gross motor or sensory deficits.

**Figure 1:**
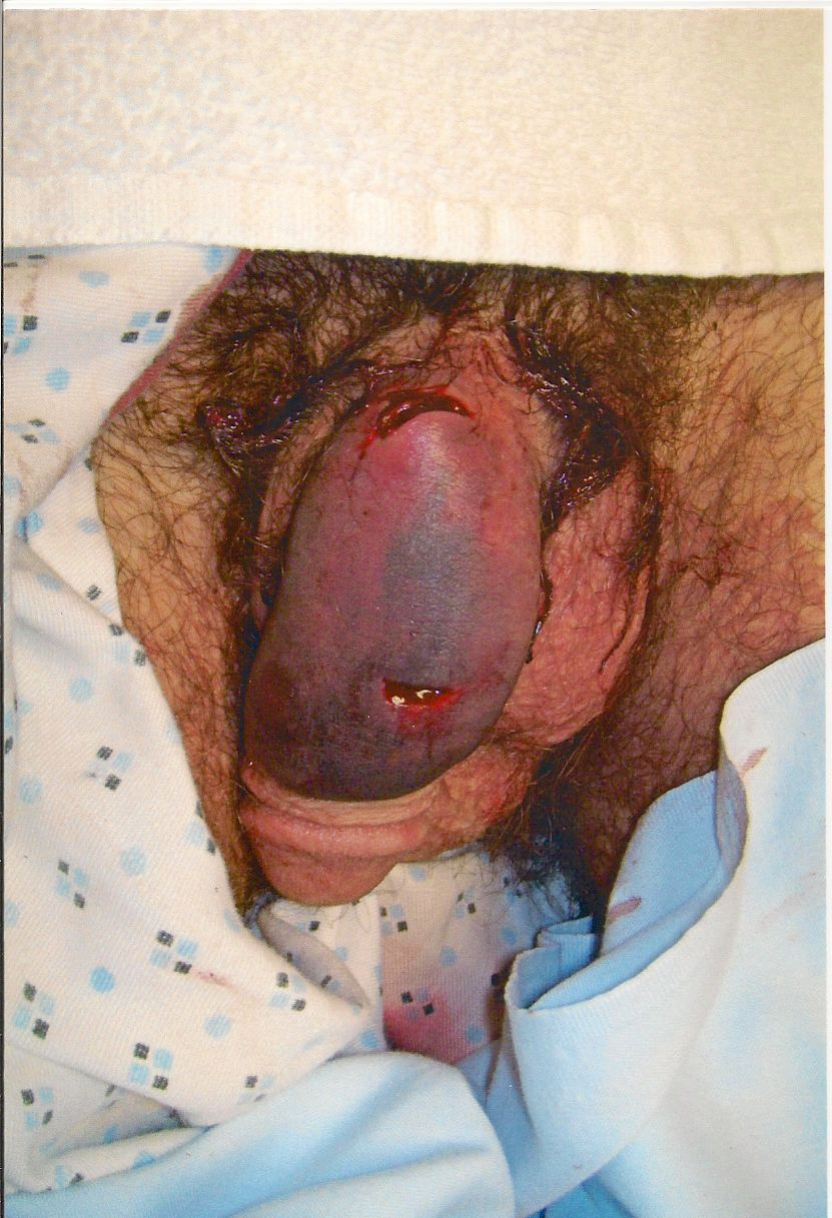
**Laceration to dorsal surface of penis**.

**Figure 2:**
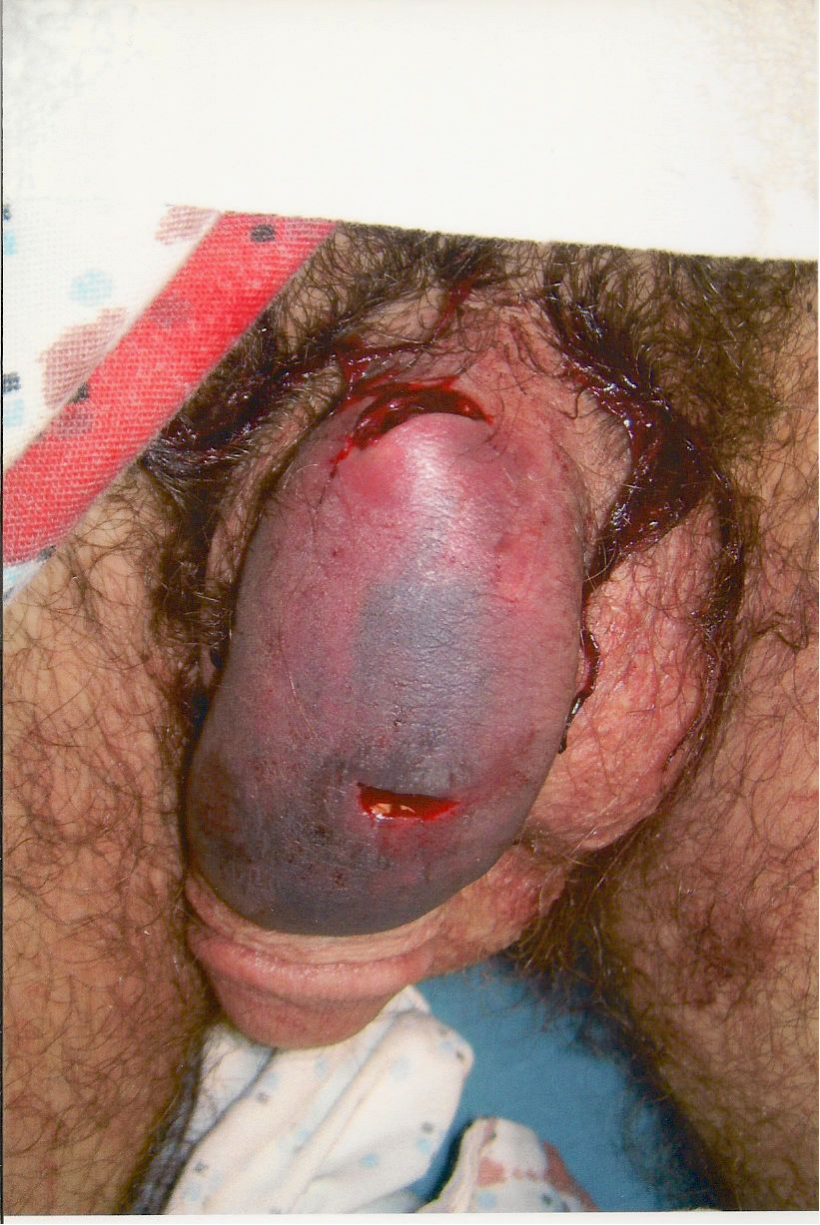
**Close-up view of lacerations**.

After removal of bandaging placed by prison medical staff, his genitourinary exam revealed an uncircumcised penis with two horizontal lacerations on the dorsal shaft, one about 1.5 cm from the base of the penis and about 1 cm in width, and the other about 1 cm from the glans and about 1 cm wide (Figures 1 and 2). There was no active bleeding to the lacerations. There was diffuse edema and ecchymosis on the dorsum of the penis with blood clots over the wounds. The wound depth was not explored at that point. There was no paraphimosis or phimosis noted. Testes were descended and nontender bilaterally with no palpable masses.

Urology was emergently consulted for surgical evaluation. Prior to Urology arrival, the patient urinated into a portable urinal, which revealed gross hematuria.

Per urological assessment, his marked penile ecchymosis and gross hematuria were suggestive of a hematoma and possible deep injury to the penis and or urethra. The patient was consented and taken emergently to the operating room for penile exploration and repair. A tetanus shot was given prior to leaving the ED.

In the operating room, the penis was degloved. It was found that the patient's two lacerations involved only the subcutaneous tissue and dartos fascia. There was no injury to Buck's fascia or to the tunica albuginea. A small subcutaneous hematoma was also evacuated from the proximal laceration. Irrigation of the wounds revealed several bleeding vessels within each wound, and they were cauterized with Bovie electrocautery. The postoperative diagnosis listed in the operative report was low velocity sharp penile injury.

The patient was subsequently brought to the surgical recovery room (PACU) in stable condition, and when fully recovered, he was discharged back to law enforcement custody. He received instructions to remove the postoperative dressings the next day, and was discharged with 5 days of cephalexin and pain medication. He was to return to the clinic in 2 weeks for a postoperative check.

## Discussion

Penile injuries, especially self-inflicted, are uncommon complaints in the ED. This case highlights a body modification practice known as "pearling" or "genital beading." In contemporary societies, this procedure is usually performed by professional body piercers where it is relatively safe and without major complications. However, "pearling" has apparently gained increasing popularity in the prison system where inmates have been doing this on their own with limited tools and knowledge of penile anatomy. This can lead to disastrous outcomes that need emergency and surgical care, as seen in this case. Other known complications due to pearling include penile abscess and pain on erection [2]. Long-term complications can include scar tissue formation causing chronic pain and/or erectile dysfunction. This is an uncommon injury in the ED, and if there is any suspicion of injury to deep penile structures, including the urethra, a urologic consultation is recommended.

## Conclusions

"Pearling," while intended to increase the sexual pleasure of partners, can cause significant morbidity to individuals themselves during object placement.

## Abbreviations

PACU: Post-Anesthesia Care Unit.

## Consent

Written informed consent was obtained from the patient for publication of this case report and any accompanying images. A copy of the written consent is available for review from the Editor-in-Chief of this journal.

## Competing interests

The authors declare that they have no competing interests.

## Authors' contributions

DN and BD saw the patient and obtained consent; DN wrote the initial report; BD and MF edited and revised the report, and added the discussion. All authors read and approved the final manuscript.
